# The Prevalence of Polyketide Synthase-Positive *E. coli* in Cystic Fibrosis

**DOI:** 10.3390/microorganisms13030681

**Published:** 2025-03-18

**Authors:** Christopher Chan, Michael Coffey, Caitlin Murphy, Isabelle McKay, Jumaana Abdu, Keerti Paida, Rachel Y. Tam, Hannah Wrigley-Carr, Bernadette Prentice, Louisa Owens, Yvonne Belessis, Sandra Chuang, Adam Jaffe, Josie van Dorst, Chee Y. Ooi

**Affiliations:** 1 School of Clinical Medicine, Discipline of Paediatrics & Child Health, UNSW Medicine & Health, University of New South Wales, Sydney, NSW 2031, Australia; z5320180@ad.unsw.edu.au (C.C.); michael.coffey@health.nsw.gov.au (M.C.); caitlinmurphy454@gmail.com (C.M.); isabellemckay-10@yahoo.com (I.M.); jumaana.abdu@health.nsw.gov.au (J.A.); h.wrigleycarr@student.unsw.edu.au (H.W.-C.); bernadette.prentice@health.nsw.gov.au (B.P.); sandra.chuang@unsw.edu.au (S.C.); a.jaffe@unsw.edu.au (A.J.); keith.ooi@unsw.edu.au (C.Y.O.); 2 Department of Gastroenterology, Sydney Children’s Hospital, Randwick, NSW 2031, Australia; 3 Department of Respiratory Medicine, Sydney Children’s Hospital, Randwick, NSW 2031, Australia; louisa.owens@health.nsw.gov.au (L.O.); yvonne.belessis@health.nsw.gov.au (Y.B.)

**Keywords:** cystic fibrosis, gastrointestinal microbiome, *E. coli*, colorectal cancer, polymerase chain reaction

## Abstract

Cystic fibrosis (CF) patients experience higher risks of colorectal cancer but the pathogenesis is unclear. In the general population, polyketide synthase-positive (pks^+^) *E. coli* is implicated in intestinal carcinogenesis via the production of colibactin; however, the relevance in CF is unknown. In this study, we investigate pks^+^ *E. coli* prevalence in CF and potential associations between pks^+^ *E. coli*, gastrointestinal inflammation, and microbiome dynamics with fecal calprotectin and 16SrRNA gene taxonomic data. Cross-sectional analysis demonstrated no difference in pks^+^ *E. coli* carriage between CF patients and healthy controls, 21/55 (38%) vs. 26/55 (47%), *p* = 0.32. Pks^+^ *E. coli* was not associated with significant differences in mean (SD) calprotectin concentration (124 (154) vs. 158 (268) mg/kg; *p* = 0.60), microbial richness (159 (76.5) vs. 147 (70.4); *p* = 0.50) or Shannon diversity index (2.78 (0.77) vs. 2.65 (0.74); *p* = 0.50) in CF. Additionally, there was no association with exocrine pancreatic status (*p* = 0.2) or overall antibiotic use (*p* = 0.6). Longitudinally, CF subjects demonstrated intra-individual variation in pks^+^ *E. coli* presence but no significant difference in overall prevalence. Future investigation into the effects of repeat exposure on risk profile and analysis of older CF cohorts is necessary to identify if associations with colorectal cancer exist.

## 1. Introduction

The gastrointestinal microbiome in cystic fibrosis (CF) demonstrates marked differences from an early age when compared to healthy subjects [1,2,3]. A significantly altered composition of the gut microbiome influences functionality [1,3], delays maturation [4,5], and contributes to a persistent inflammatory state [4]. An improved life expectancy in CF patients, resulting from therapeutic advancements, has contributed to rising diagnoses of concomitant disorders, including gastrointestinal malignancy [6]. Individuals with CF experience around 10 times the risk of developing colon cancer, coupled with earlier onset of cancer development when compared to the general population [6,7,8]. Crucially, dysbiosis and increased inflammation are both a feature of the CF gut microbiome [4,9] and implicated in the development of colorectal cancer (CRC) [10], suggesting these factors may influence CF-related CRC.

The normal gut microbiome becomes established from the age of four and remains stable into adulthood [11]. Given that the altered intestinal milieu in CF is established from early childhood, there is reason to consider that the physiological steps that prime CF patients to develop CRC may be identifiable in childhood. Children with CF (cwCF) demonstrate significantly increased abundances of *Escherichia coli* (*E. coli*) in the gut [1,12]. While *E. coli* is commensal, some strains may cause pathology [13], particularly in vulnerable gastrointestinal environments such as those of cwCF. *E*. *coli* derived from the B2 phylogenetic group can produce colibactin, a genotoxic chemical compound, through expression of a polyketide synthase (pks) gene locus [14].

The pathogenicity of colibactin is believed to stem from the alkylation of DNA on adenine residues and induction of double strand breaks [15,16]. Moreover, a distinct mutational signature attributed to colibactin has previously been identified in a subset of CRC patients following whole genome sequencing [17]. Colibactin has also demonstrated causative links to microbial dysbiosis and intestinal inflammation [18,19]. Taken together, this suggests pks^+^ *E. coli* may be overrepresented in CF. However, no studies have investigated a potential role for colibactin in CF-related gut changes. The isolation of colibactin for research purposes is difficult due to chemical instability and small production quantities [20]. To counter this, previous research methods have focused on the pks gene island and its regulation of colibactin production to characterise genotoxic ability and therapeutic potential [21].

In this study, we aimed to investigate differences in pks island expression, as a proxy for colibactin, between cwCF and HC (healthy controls) and within cwCF over time. We also investigated inflammation and microbial diversity within the CF gut as well as potential associations between *pks* expression, gut inflammation, and dysbiosis.

## 2. Materials and Methods

### 2.1. Study Design

CwCF were recruited from the CF clinic at Sydney Children’s Hospital (SCH) Randwick, an Australian tertiary paediatric hospital, as part of the ‘Evaluating the Alimentary and Respiratory Tracts in Health and Disease’ (EARTH) research programme, beginning in 2018 [22]. HC were recruited through advertisement, word-of-mouth, and from outpatient clinics (e.g., healthy children from the fracture clinic). Eligible patients were aged between 0 and 18 years of age, provided informed parental written consent, and either fulfilled the United States Cystic Fibrosis Foundation consensus diagnostic criteria (CF group) or suffered no chronic health condition (HC group). Furthermore, patients did not modify their treatment or pharmacological regimens, including CFTR modulator and antibiotic therapy, for the purposes of this study. Conversely, patients were excluded if they had multiple chronic diseases, were unable to meet the study requirements (sample collection and survey completion), or if their guardian was unable to speak or understand English. Further inclusion criteria into the EARTH programme have been previously outlined in the EARTH protocol [22].

The study involved 55 children with CF and 55 HC [22]. Overall, 23 CF patients also met the criteria for longitudinal analysis, requiring three consecutive stool samples, collected at separate timepoints approximately six months apart. The EARTH research programme was approved by the South-Eastern Sydney Area Health Service, Human Research Ethics Committee, Sydney, Australia. Ethics number: HREC/18/SCHN/26 and 2019/ETH05421.

### 2.2. Dietary Surveys and Analysis

Dietary intakes were quantified by the Australian Child and Adolescent Eating Survey (ACAES), a semi-quantitative food frequency questionnaire measuring intakes over the preceding 6 months (validated for Australian participants aged 2 years or older) (University of Newcastle, Australia) [23] according to the methods outlined in the EARTH protocol [22]. From the dietary intake survey results, 5 dietary variables (total fat, saturated fat, meat, total sugar, and total fibre) were selected for analysis in our study, based on previous associations with CRC risk [24,25,26]. Children under 2 years of age underwent a dietitian-administered 24-h food recall, measuring intakes over the preceding day. Nutrient intake data from the recalls was extracted using FoodWorks (v9) and the following databases: AusFoods 2017 and AusBrands 2017 (Xyris Software, Brisbane, Australia). A registered dietitian inspected the recalls for completeness and plausibility.

### 2.3. DNA Extraction and Sequencing

DNA was extracted from homogenised stool samples using the QIAamp DNA Mini Kit (Qiagen, Germantown, MD, USA), as utilised by Nielsen et al. [27] and in accordance with manufacturer instructions. The 16s rRNA gene amplicon sequencing was performed at the Ramaciotti Centre for Genomics, University of New South Wales, on the Illumina MiSeq platform (v3, 2 × 300 bp). Sequences were quality filtered, clustered into unique zero-distance operation taxonomic units (zOTUs), and classified as described by Coffey et al. [1].

### 2.4. Fecal Calprotectin

Fecal calprotectin was measured to assess presence of intestinal inflammation using the BÜHLMANN fCAL ELISA kit (BÜHLMANN Laboratories AG, Schönenbuch, Switzerland), as demonstrated by Garnett et al. [28]. This method has been previously validated for CF cohorts [29].

### 2.5. Identification of E. coli and pks-Producing E. coli

A polymerase chain reaction (PCR) was performed to screen fecal DNA samples for pks^+^ *E. coli*. The detection of pks^+^ bacterial DNA involved the amplification of a 283 bp sequence in the gene *clbB*, part of the pks island, with the following primer set: forward primer 5′-GCGCATCCTCAAGAGTAAATA-3′ and reverse primer 5′-GCGCTCTATGCTCATCAACC-3′ [30]. To confirm the presence of *E. coli* specific pks^+^, samples were also screened for *E. coli*, via amplification of a primer set universal for the *E. coli* specific gene *UidA*: forward primer 5′-AAAACGGCAAGAAAAAGCAG-3′ and reverse primer 5′-ACGCGTGGTTACAGTCTTGCG-3′ [31]. Samples were considered as pks^+^ *E. coli* positive if they were positive for the *clbB* and *UidA* gene cluster on PCR.

Invitrogen^®^ Super Mix (Invitrogen/Thermo Fisher Scientific, 10572014, Waltham, MA, USA) and Invitrogen^®^ Platinum™ II Hot-Start Green PCR Master Mix (2X) (Invitrogen/Thermo Fisher Scientific, 14001014, Waltham, MA, USA) were used for the PCR reactions. DNA from pks^+^ *E*. *coli*, isolated from Mutaflor^®^ *Escherichia coli* strain Nissle 1917 (Mutaflor, 123456, Adelaide, Australia), was used as a positive control, while a negative control was prepared by substituting the DNA with nuclease free water. The final volume of the PCR reactions was 25 μL, consisting of 22.5 μL of Invitrogen^®^ Super Mix or Invitrogen^®^ Platinum™ II Hot-Start Green PCR Master Mix (2X), 0.5 μL of each primer (forward and reverse), and 1.5 μL of the isolated stool sample DNA. Amplification was performed using a SimpliAmp™ Thermal Cycler (Applied Biosystems/Thermo Fisher Scientific, Waltham, MA, USA) (94 °C for 2 min, followed by 35 cycles of 94 °C for 15 s, 58.5 °C for 15 s, 72 °C for 60 s, then a final holding step at 4 °C). The products were visualized and evaluated on a prepared 1% agarose gel containing a SYBR^®^ Safe DNA gel stain (Invitrogen/Thermo Fisher Scientific, S33102, Waltham, MA, USA).

### 2.6. Statistical Analysis

We compared age and gender differences between CF and HC cohorts using Student’s *t*-test and Fisher’s Exact test, respectively. Any statistical differences in the proportions of pks^+^ *E. coli* prevalence between groups were then determined using Pearson’s chi-squared test for independence. Next, alpha diversity was calculated using two metrics in the vegan R package: Richness—a measure of unique zOTUs—and Shannon diversity index (H’)—a measure of species abundance and evenness. To identify the effect of *clbB* positive gene status on alpha diversity, the mean and standard deviation (SD) of these metrics were calculated through paired Student’s *t*-tests. Next, Fisher’s Exact test was utilised to determine if a relationship exists between *clbB*-positive gene status and exocrine pancreatic status and overall antibiotic use. A Student’s *t*-test and Mann–Whitney U test were then utilised to assess the relationship between *clbB* prevalence and selected dietary metrics after a Shaprio–Wilk test for normalcy was utilised to determine the distribution of data. Linear mixed models were then constructed to control for age and gender when assessing potential differences in alpha diversity with longitudinal data. Finally, permutational multivariate analysis of variance (PERMANOVA) tests (permutations = 1000) were utilised to test if beta diversity was significantly different between groups (‘CF versus HC’ and ‘Timepoint 1’ versus ‘Timepoint 2’ versus ‘Timepoint 3’) using the vegan function adonis2 [32]. Statistical significance was reported at the 5% significance level throughout. All statistical analysis was performed in RStudio v2023.03.0+386. Graphs were generated using ggplot2 in RStudio [33].

## 3. Results

### 3.1. Demographics

Both the CF and HC group were comprised of 26 males (47%). The mean (SD) age of CF patients and HC was 7.7 years (5.1) and 7.8 years (5.0), respectively, *p* = 0.17. Among the CF patients, 48 patients (87%) were pancreatic insufficient. Anthropometric z scores for our CF cohort were also calculated, based on WHO regression data for the general paediatric population, and are presented in Table 1 (part 1A). The CF subgroup (n = 23) available for longitudinal analysis was comprised of 10 males and 13 females. The mean age at each timepoint was 7.0 years (4.2), 7.8 years (4.2), and 8.8 years (3.9) for timepoints 1, 2, and 3, respectively. Of this subset, 19 patients (83%) were pancreatic insufficient (Table 1 (part 1B)). Microbial alpha diversity and phylogeny-based beta diversity were calculated with a dataset subsampled to 33,839 and 13,497 sequences per sample for baseline and longitudinal analysis, respectively.

### 3.2. Microbiome Diversity and Inflammation

**Figure 1:**
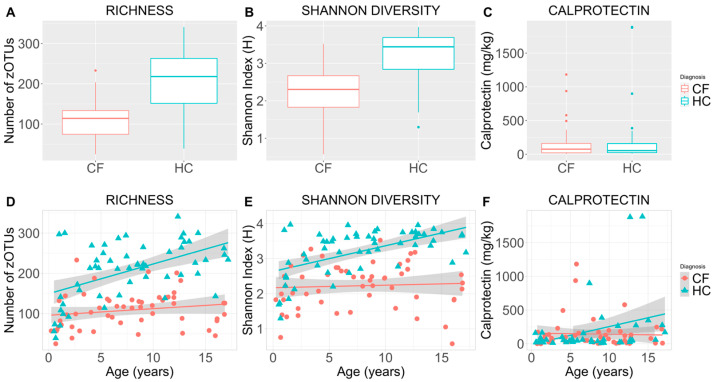
Alpha diversity indices and fecal calprotectin for cross-sectional comparison of children with cystic fibrosis versus healthy controls. Boxplots depict microbial richness (number of zOTUs)) (**A**), Shannon diversity (Shannon Index (H units)) (**B**), and the concentration of fecal calprotectin (mg/kg) (**C**) in fecal samples of the cystic fibrosis and healthy control cohorts. The central boxplot line indicates the median of the focus group, with the top and bottom values indicating the 1st and 3rd quartile. Scatterplots illustrate sample microbial richness (**D**), Shannon diversity (**E**), and fecal calprotectin (**F**) in the cystic fibrosis and healthy control cohorts with respect to age (years). Coloured lines indicate the cohort means, while shaded regions indicate 95% confidence intervals constructed from generalised linear models (**D**–**F**). CF = cystic fibrosis, HC = healthy controls, zOTU = zero-distance operation taxonomic unit, calprotectin = a measure of intestinal inflammation.

The mean (SD) microbial richness was significantly decreased in CF patients compared to HC (108 (45) versus 207 (71); *p* < 0.001) (Figure 1A,D). The Mean Shannon diversity was also significantly decreased among CF patients compared to HC (2.2 (0.65) versus 3.2 (0.65); *p* < 0.001) (Figure 1B,E). When accounting for age, Shannon diversity remained significantly lower relative to HC (*p* = 0.001). No difference in either alpha diversity indices was observed between males and females. CF participants had significantly elevated mean calprotectin levels, indicative of increased intestinal inflammation relative to HC (232 (402) mg/kg vs. 64 (73) mg/kg; *p* < 0.003). This disparity remained unchanged when controlling for age (Figure 1C,F).

### 3.3. Prevalence of pks^+^ E. coli at Baseline

The presence of *clbB* was identified in 21 CF patients and 26 HC (Table 2). Of the 47 individuals positive for the *clbB* gene, four patients (three CF, one HC) were negative for the *UidA* gene cluster. Pearson’s chi-squared test for independence demonstrated no significant difference in the proportion of *clbB* positive subjects between CF and HC (*p* = 0.4, df = 1). In our CF cohort, the presence of *clbB* was also not correlated with any significant differences in exocrine pancreatic status (*p* = 0.2), antibiotics use (*p* = 0.6), mean calprotectin concentration (*p* = 0.6), microbial richness (*p* = 0.5), or Shannon diversity (*p* = 0.5) (Table 3). With regard to diet, there was no significant correlation observed between *clbB* prevalence and dietary factors of diet, total fat, saturated fat, meat, total sugar, and total fibre (Table S3).

Table 2 depicts a two-way frequency table of *clbB* gene status (positive versus negative) against diagnosis (cystic fibrosis versus healthy controls) from cross-sectional baseline analysis of fecal DNA samples. The *clbB* gene was screened for, using PCR, to determine the prevalence of the *pks* island. Pks = polyketide-synthase.

Table 3 depicts the mean and *p*-value following Student’s *t*-test analysis of correlations between *clbB* prevalence and microbial diversity indices (Shannon diversity and microbial richness) or calprotectin. Pks = polyketide-synthase, Microbial richness = a measure of unique zOTUs, Shannon diversity = a measure of species abundance and evenness, Calprotectin = a measure of intestinal inflammation, SD = standard deviation.

### 3.4. Prevalence of pks^+^ E. coli in a Longitudinal Analysis of the CF Cohort

The chi-squared test for independence revealed no significant differences in *clbB* prevalence across timepoints (*p* = 0.4, df = 2), even when accounting for age (*p* = 0.6) and antibiotics usage (*p* = 0.18). However, only 7/23 (30%) subjects were consistent in their pks^+^ *E. coli* status across all three timepoints (positive or negative). The fluctuation in pks^+^ *E. coli* prevalence across each timepoint is presented in full in Table 4. Furthermore, 12/69 (17%) of the total samples were negative for *UidA*. A linear mixed model was used to investigate the effects of age, gender, and sample timepoint on alpha diversity indices, with Patient ID included as a random effect to account for repeated measures. There was no significant difference in alpha diversity parameters (microbial richness, Shannon diversity or Chao1) between sample timepoints (variance = 412.9, 0.15, 344.9; *p* = 0.9, 0.2, 0.9 respectively) and no effect of gender. Alpha diversity parameters did increase with age, but this was only a significant predictor for Chao1 (β = 4.07, SE = 1.75, t = 2.32, *p* = 0.03) (Table 5).

## 4. Discussion

This study is the first of its kind to investigate colibactin-producing pks^+^ *E. coli* in the CF gut. We identified no evidence that suggests pks^+^ *E. coli* prevalence is increased in cwCF. Furthermore, pks^+^ *E. coli* prevalence was not linked to differences in exocrine pancreatic status, overall antibiotic use, calprotectin concentration, gut microbial richness, Shannon diversity index, or diet within cwCF. While many cwCF demonstrated fluctuation in their pks^+^ *E. coli* status across timepoints, there was no significant difference overall. We also identified significant reductions in microbial diversity of the CF gut and significant differences in bacterial composition when compared to HC. Fecal calprotectin was significantly higher in cwCF compared to HC; however, within cwCF, there were no differences in calprotectin concentrations over time.

Our results suggest that increased abundances of pks^+^ *E. coli* are not a major contributor to alterations in gut richness or diversity for cwCF. Furthermore, we did not identify any significant relationship between pks^+^ *E. coli* status and inflammation or gut microbial diversity. Alpha and beta diversity provide an important characterization of the gut microbiota; however, they are unable to characterize DNA changes, which occur following exposure to genotoxic bacteria like pks^+^ *E. coli* or its metabolic products, such as colibactin. Specific mutational signatures associated with colibactin, such as single base substitution (SBS)-88 and short insertion and deletion (indel)-18 signatures, have been previously identified through in-vitro organoid studies [17]. Future metagenomics analysis of CF patient microbiomes may reveal if similar mutational signatures exist in this cohort.

The significantly lower gut microbial richness and diversity in cwCF and increased fecal calprotectin mirror the findings of previous CF gut microbiome studies [3,27,34]. Our CF cohort exhibited alterations in over 70 genera, most notably demonstrating increases in the genera *Fusobacterium* and *Enterococcus*. Strains of these genera have previously demonstrated individual links to CRC development [35]. Furthermore, it is unknown if the presence of other specific bacteria influences the activity of pks^+^ *E. coli* in CF. A previous study of recurrent *Clostridioides difficile* infection demonstrated eradication of pks^+^ *E. coli*, in patients with initially detectable levels, following fecal microbial transplantation [36]. Such distinct changes in microbiome are comparatively different in a CF context, and inferences on causality in CF remain elusive.

Our analysis identified no significant differences in the proportion of pks^+^ *E. coli* cwCF over time, along with no significant temporal changes in microbial alpha diversity or gut inflammation. However, 16 individuals experienced fluctuations in their pks^+^ *E. coli* status across successive timepoints, indicating a high degree of temporal instability. Furthermore, only three patients maintained positive pks^+^ *E. coli* status across all three timepoints. This fluctuation has not previously been reported in a CF cohort. However, one study of an IBD cohort identified greater temporal microbiota instability when compared to HC, particularly with changes in disease activity [37]. It remains unclear whether the persistence of pks^+^ *E. coli* is required for a genotoxic effect or if the cumulative influence of transient repeat exposures is satisfactory to produce carcinogenesis. Given the high variability we identified in pks^+^ *E. coli* status, the concept of repeat transient exposures could be relevant in CF-related CRC.

We identified the presence of pks in four samples (three CF, one HC) that did not have *E. coli*, suggesting the presence of non-*E. coli* pks^+^ bacteria. Certain strains of *Klebsiella pneumoniae*, as well as *Pseudovibrio* species, have previously been identified as pks^+^ [38]. However, these studies were conducted either in vitro or concerned non-human species. Thus, their clinical significance remains undetermined. We also identified one subject (CF9) who experienced undetectable *UidA* and *clbB* levels across all three timepoints in longitudinal analysis. Given that the greater abundance of *E. coli* in CF compared to the general population is well established [1,12], these results were unexpected but highlight the high level of variability and complexity of CF microbiota and pathophysiology.

While the implications of pks^+^ *E. coli* on colorectal carcinogenesis have been previously reported in non-CF populations [30,39], a comparison of pks^+^ *E. coli* abundances between studies is markedly different. It is also important to note that pks^+^ *E. coli* represents one of many distinct pathogenic pathways toward carcinogenesis in CF. Other non-pks containing organisms, including *Fusobacterium nucleatum*, *Bacteroides fragilis*, *Enterococcus faecalis* and *Salmonella* sp., have been associated with CRC across cell culture and pre-clinical and observational human studies. Furthermore, the cystic fibrosis transmembrane conductance regulator (CFTR) has previously demonstrated anti-tumorigenic properties [40]. The role of other familial-inherited mutations in CF, such as familial adenomatous polyposis and Lynch syndrome, should also be considered. The high-energy high-fat CF legacy diet utilised in CF nutrition management has also been shown to influence GI microbial composition [41] and has links to GI inflammation. Moreover, the Western-style diet, featuring high-fat and high-sugar products and low levels of fibre, is conducive to higher rates of CRC containing pks^+^ *E. coli* [42].

Some limitations of our current study should be considered. While use of the *clbB* gene as a proxy for the pks island has been validated in previous studies, this gene represents one of multiple within the pks gene cluster. PCR also limited our research to presence/absence analysis. Quantitative PCR would allow examination of longitudinal changes in gene expression and when these changes occur. While it has previously been utilised for colibactin analysis, this was not in a CF cohort [43]. Sample specific issues for DNA extraction could also influence pks prevalence, including low DNA extraction efficiency, DNA degradation, low concentrations of PKS, and inhibition of PCR by other stool substances. Furthermore, our study accounted for the demographic co-variates of age and gender, along with other potential co-variables including antibiotic use and key dietary variables. Yet, other complex interactions and hereditary factors remain. These include the contribution of the CF legacy diet to inflammation and CRC risk and the impact of specific antibiotic and medication combinations, such as the therapeutic complexity involved in CF management. Future studies should investigate the potential role of these factors, in particular the CF legacy diet, in CRC development.

## 5. Conclusions

Taken together, our findings suggest that pks^+^ *E. coli* is unlikely to directly contribute to the increased gut inflammation and dysbiosis that develops early in cwCF. As our study was undertaken in cwCF, future investigation of the adult CF population is required to understand how pks and colibactin may influence inflammation and colorectal carcinogenesis later in life. The characterisation of pks in older CF patients with polyp formation or diagnosis of CRC would further this understanding. Given the greater abundance of bacteria with known carcinogenic links, along with the temporal variability we identified in pks^+^ *E. coli*, future research on the CF gut microbiome should also consider the cumulative impact of multiple genotoxic species and the long-term effects that recurrent colonisation of these genotoxic species may have.

## Figures and Tables

**Table 1:** Demographics for cross-sectional comparison (1A) and longitudinal analysis (1B).

1A: Baseline		Cystic Fibrosis Patients		Healthy Controls	*p*-Value		95% CI
Number of participants		55		55			
Male sex, n (%)		26 (47%)		26 (47%)	1		
Mean age in years (SD)		7.66 (5.1)		7.78 (5.0)	0.17		(−0.29, 0.05)
Exocrine pancreatic status		**Cystic fibrosis patients**					
	Pancreatic sufficient (%)	7 (13%)					
	Pancreatic insufficient (%)	48 (87%)					
Anthropometrics							
	Mean weight z-scores (SD)	−0.24 (1.23) 0.00 (1.01) 0.39 (0.71)					
	Mean height z-scores (SD)						
	Mean BMI z-scores (SD)						
**1B: Longitudinal**							
Number of participants		23					
Male sex, n (%)		10 (38%)					
Mean age at specified timepoint in years (SD)		T1 7.0 (4.2)	T2 7.8 (4.2)			T3 8.8 (3.9)	
Exocrine pancreatic status							
	Pancreatic sufficient (%)	4 (17%)					
	Pancreatic insufficient (%)	19 (83%)					

Cross-sectional analysis involved cystic fibrosis patients (n = 55) and healthy controls (n = 55) (**1A**). Longitudinal analysis involved cystic fibrosis patients only (n = 23) (**1B**). Fisher’s Exact test was utilised to determine gender differences between cystic fibrosis patients and healthy controls. Anthropometric z-scores were calculated using WHO regression data for the general paediatric population. A Student’s *t*-test was utilised to determine if significant differences in age exist between CF and HC. CF = cystic fibrosis, HC = healthy controls, T1 = Timepoint 1, T2 = Timepoint 2, T3 = Timepoint 3, n = number of samples, CI = confidence interval, SD = standard deviation.

**Table 2:** Distribution of the *pks* island in cross-sectional analysis.

	Diagnosis		
*clbB* Status	Cystic Fibrosis	Healthy Controls	Total
Positive	21	26	47
Negative	34	29	63
Total	55	55	110

**Table 3:** Correlation between *clbB* gene prevalence (as a proxy for pks island prevalence) and alpha diversity and calprotectin in cystic fibrosis patients.

		Mean (SD)	*p*-Value
**Calprotectin (mg/kg)**			
	*clbB* positive	124 (154)	0.6
	*clbB* negative	158 (268)	
**Shannon diversity (Shannon Index (H))**			
	*clbB* positive	2.78 (0.77)	0.5
	*clbB* negative	2.65 (0.74)	
**Microbial richness**			
	*clbB* positive	159 (76.5)	0.5
	*clbB* negative	147 (70.4)	

**Table 4:** Longitudinal carriage of pks^+^ *E. coli* in children with cystic fibrosis over successive timepoints.

	Timepoint 1	Timepoint 2	Timepoint 3
CF1	+		
CF2	+		
CF3	-	-	-
CF4	+	+	+
CF5	+	-	+
CF6	-	-	-
CF7	-	+	-
CF8	-	-	+
CF9			
CF10		-	
CF11	-	-	+
CF12	+	+	+
CF13	+	-	+
CF14	-	+	-
CF15	-		+
CF16	+	+	
CF17	-	-	+
CF18	+	-	+
CF19	+	+	+
CF20	-	+	-
CF21	+	-	
CF22	+	-	+
CF23	-	-	+

The prevalence of pks^+^ *E. coli* was determined by being positive for both the *clbB* and *UidA* gene cluster on PCR. Here, ‘+’ indicates that pks^+^ *E. coli* is present in the sample, ‘-’ indicates that pks^+^ *E. coli* is absent in the sample, and a grey space indicates that *E. coli* was undetectable in this sample following *UidA* gene PCR. Each row corresponds to one de-identified cystic fibrosis patient involved in the longitudinal analysis. Samples were collected across three timepoints, spaced roughly six months apart. pks^+^ *E. coli* = polyketide synthase-positive *Escherichia coli*.

**Table 5:** Results of linear mixed model analysis, investigating the effects of age, gender, and sample timepoint on alpha diversity indices Richness, Shannon diversity, and Chao1.

		*p*-Value	Standard Error	Estimate	T-Value
**Richness**					
	*Timepoint 2*	0.9	10.8	1.79	0.16
	*Timepoint 3*	0.135	11.1	16.9	1.52
	*Sex*	0.5	12.4	8.17	0.66
	*Age*	0.0589	1.52	3.04	2.00
	*Variance of intercepts*	412.9	20.3		
	*Variance of residuals*	1330.1	36.5		
**Shannon Diversity**					
	*Timepoint 2*	0.178	0.16	−0.22	−1.37
	*Timepoint 3*	0.5	0.17	0.10	0.61
	*Sex*	0.2	0.21	0.27	1.29
	*Age*	0.6	0.025	0.013	0.51
	*Variance of intercepts*	0.154	0.0393		
	*Variance of residuals*	0.295	0.543		
**Chao1**					
	*Timepoint 2*	0.9	14.5	0.49	0.034
	*Timepoint 3*	0.2	14.8	17.7	1.20
	*Sex*	0.4	14.2	10.4	0.73
	*Age*	0.0306 *	1.75	4.07	2.32
	*Variance of intercepts*	344.9	18.6		
	*Variance of residuals*	2381.7	48.8		

*p*-values indicate the significance of the relationship between Timepoint 1 and the target variable. * = a statistically significant value.

## Data Availability

The original contributions presented in this study are included in the article. Further inquiries can be directed to the corresponding author.

## Supplementary Materials

The following supporting information can be downloaded at: https://www.mdpi.com/article/10.3390/microorganisms13030681/s1, Table S1. Bacterial taxa that demonstrate significantly different abundance in cystic fibrosis patients compared to healthy controls at each taxonomic rank. Table S2. Bacterial taxa at each taxonomic rank, which demonstrate significantly different abundance in the cystic fibrosis gut longitudinally. Table S3. Correlations between selected dietary metrics and *clbB* prevalence in cross-sectional CF analysis. Figure S1. PCR products following agarose gel electrophoresis for detection of *clbB* in a sample of CF patients. Figure S2. PCR products following agarose gel electrophoresis for optimisation of *clbB* gene primers. In total 11 PCR reactions were prepared with our pks^+^ *E. coli* positive control, isolated from Mutaflor^®^ *Escherichia coli* strain Nissle 1917 (Mutaflor), along with one negative control prepared with nuclease free water. PCR reactions were amplified with forward and reverse *clbB* primers in Invitrogen^®^ Super Mix. A 1 kB DNA ladder was run alongside samples to ensure the correct product was obtained. Reaction tubes were amplified across 4 different annealing temperatures (3 × 55 °C, 3 × 56.5 °C, 3 × 58.5 °C, and 2 × 60 °C) using four SimpliAmp™ Thermal Cyclers (Applied Biosystems/Thermo Fisher Scientific, Waltham, MA, USA) set at each temperature. From the result, a determination on the optimal annealing temperature (58.5 °C) was made. PCR = polymerase chain reaction, pks^+^ *E. coli* = polyketide synthase-positive *Escherichia coli*, DNA = deoxy-ribonucleic acid. Figure S3. Relative abundance (%) of the most abundant bacterial phyla in the cystic fibrosis gut versus healthy controls. Figure S4. Relative abundance (%) of the most abundant bacterial phyla in the cystic fibrosis gut over time. Figure S5. Longitudinal microbial richness of cystic fibrosis patients. Figure S6. The proposed pathogenic factors associated with the development of an altered gastrointestinal microbiota in cystic fibrosis. The composition of the gut microbiota in cystic fibrosis is abnormal, where CFTR dysfunction contributes to reduced bicarbonate secretion and subsequent reductions in pH. Furthermore, reduced bicarbonate contributes to the development of hyper-viscous mucus, impairing intestinal motility. Regular antibiotics use and a diminished innate immune response against pathogenic bacteria may also contribute. The cumulative impact of these changes is an altered gastrointestinal milieu is characterised most notably the raised abundance of *Escherichia coli*. CFTR = cystic fibrosis transmembrane conductance regulator. *E. coli* = *Escherichia coli*. Adapted from “Epithelial Mucosa”, by BioRender.com (2023). Retrieved from https://app.biorender.com/biorender-templates. Figure S7. Colibactin induces colon cell mutation. During infection with pks^+^ *E. coli*, where pks^+^ *E. coli* comes into direct contact with the host epithelium, colibactin molecules migrate to the host cell nucleus to initiate DNA breaks. Infection with pks^+^ *E. coli* also facilitates cell-cycle arrest, causing a senescence related secretory phenotype, which stimulates growth factor release into the tumour microenvironment [20]. These combined processes contribute to colon cell mutagenesis. pks^+^ *E. coli* = polyketide synthase-positive *Escherichia coli*, DNA = deoxy-ribonucleic acid, SP = senescence-associated secretory phenotype, GF = growth factor. “Created with BioRender.com”. Figure S8. Relative abundance (%) of the top 10 most abundant bacterial genera in the cystic fibrosis gut versus healthy controls. Samples have been de-identified and assigned an ID based on diagnosis (CF1–CF55, HC 1–55). The diagnosis of each sample is indicated above its respective column. Relative abundance was calculated as the abundance of a single zOTU, divided by the sum of total zOTU abundances. The Nucleotide Basic Local Alignment Search Tool (BLASTn) in the SILVA 123 ribosomal RNA (rRNA) database was utilised to align zOTUs to specific genera. This graph was generated using ggplot2 in R [33]. Relative abundance = the rarity of a particular species relative to other species within the community, CF = cystic fibrosis, HC = healthy control, zOTU = zero-distance operation taxonomic unit. Figure S9. Longitudinal analysis of alpha diversity indices and fecal calprotectin in children with cystic fibrosis. Boxplots depict fecal sample richness (number of zOTUs) (A), Shannon diversity (Shannon Index (H)) (B), and the concentration of fecal calprotectin (mg/kg) (C) across 3 consecutive timepoints, spaced roughly 6 months apart. Scatterplots illustrate sample richness (D), Shannon diversity (E), and fecal calprotectin (F) across timepoints with respect to age (years). Coloured lines indicate the cohort means, while shaded regions indicate 95% confidence intervals constructed from generalised linear models (D–F). calprotectin = a measure of intestinal inflammation, zOTU = zero-distance operation taxonomic unit. Figure S10. Longitudinal analysis of relative abundance for the 10 most abundant bacterial genera in cystic fibrosis patients. Samples have been de-identified and assigned an ID (CF1–CF23). Samples with the same ID correspond to the same patient. The timepoint of each sample is indicated above its respective column. Relative abundance was calculated as the abundance of a single zOTU divided by the sum of total zOTU abundances. The Nucleotide Basic Local Alignment Search Tool (BLASTn) in the SILVA 123 ribosomal RNA (rRNA) database was utilised to align zOTUs to specific genera. This graph was generated using ggplot2 in R [33]. Relative abundance = the rarity of a particular species relative to other species within the community, CF = cystic fibrosis, zOTU = zero-distance operation taxonomic unit. Figure S11. Bray–Curtis and binary Bray–Curtis analysis for cross-sectional comparison of children with cystic fibrosis versus healthy controls. Figure S12. Bray–Curtis analysis for longitudinal comparison of children with cystic fibrosis at first, second, and third sample timepoints.
