# Supplementary material for: The Prevalence of Polyketide Synthase-Positive E. coli in Cystic Fibrosis
Source: Microorganisms. 2025 Mar 18;13(3):681. doi: 10.3390/microorganisms13030681 (PMC11944406; doi:10.3390/microorganisms13030681)
Supplement: Supplementary file 1 [file microorganisms-13-00681-s001.zip › microorganisms-3477440-supplementary.pdf]

Table S1. Bacterial taxa that demonstrate significantly different abundance in cystic fibrosis patients compared to healthy controls at each taxonomic rank.

Phylum	Class	Order	Family	Genus
Actinobacteria ↑	Actinobacteria ↑	Corynebacteriales ↑	Nocardiaceae ↑	Rhodococcus ↑
				Adlercreutzia ↓
				f. Corynebacteriaceae ↑
			Coriobacteriaceae	f. Coriobacteriaceae ↓
Bacteroidetes	Flavobacteria ↑			Capnocytophaga ↑
				Gordonibacter ↓
		Bacteroidales	Porphyromonadaceae ↓	Barnesiella ↓
				Butyricimonas ↓
				Butyricicoccus ↑
				f_Bacteroidales S24–7 group ↓
				Odoribacter ↓
			Muribaculaceae ↓	
			Rikenellaceae ↓	Alistipes ↓
Deinococcus. Thermus	Deinococci ↑			
Euryarcheota ↓	Methanobacteria ↓		Methanobacteriaceae ↓	Methanobrevibacter ↓
Firmicutes	Bacilli ↑	Bacillales	Family XIII ↓	Family XIII ↓
		Clostridiales ↓	Christensenellaceae ↓	Eubacterium ↓
				Christensenellaceae ↓
			Family XI	Peptoniphilus ↑
			Peptococcaeceae ↓	Acetanobacterium ↓
				f. peptococcaceae ↓
				Peptococcus ↓
			Defluviitaleaceae ↓	Defluviitaleaceae UCG-011 ↓
			Ruminococcaceae ↓	Anaerofilum ↓
				Anaerotruncus ↓
				Butyricicoccus ↑
				Candidatus Soleaferrea ↓
				f. ruminococcaceae ↓
				Hydrogenoanaerobacterium↓
				Faecalibacterium ↓
				Intestinimonas ↓
				Oscillibacter ↓
				Oscillospira ↓
				Ruminiclostridium ↓
				Ruminococcaceae.UCG ↓
				Ruminococcus ↓
				Subdoligranulum ↓
			Lachnospiraceae	Anaerostipes ↑
				Coprococcus ↓
				Dorea ↓
				Eisenbergiella ↓
				Fusicatenibacter ↓
				Lachnospira ↓
				Lachnospiraceae.UCG ↓
				Lactonifactor ↓
				Marvinbryantia ↓
				Pseudobutyrivibrio ↓

				Roseburia ↓
				Tyzzerella ↑
			Clostridiaceae ↑	Clostridium sensu stricto. 1 ↑
				Sarcina ↑
		Thermoanaerobacteriales	Thermoanaerobacteriaceae ↓	Gelria ↓
	Clostridia ↓		Peptostreptococcaceae ↑	Peptoclostridium ↑
	Erysipelotrichia ↓	Lactobacillales ↑	Enterococcaceae ↑	Enterococcus ↑
			Erysipelotrichaceae ↓	Asteroleplasma ↓
				Dielma ↓
				Erysipelotrichaceae UCG-003 ↓
				Holdemania ↓
				Incertae Sedis ↓
			Lactobacillaceae ↑	Lactobacillus ↑
			Veillonelaceae	Dialister ↓
				Megasphaera ↑
				Veillonella ↑
Fusobacteria ↑	Fusobacteriia ↑	Fusobacteriales ↑	Fusobacteriaceae ↑	Fusobacterium ↑
Lentisphaerae ↓	Lentisphaeria ↓	Victivallales ↓	Victavallaceae ↓	Victavallis ↓
Proteobacteria ↑	Alphaproteobacteria ↓	Rhodospiralles ↓	Rhodospirillaceae ↓	Thalassospira ↓
	Betaproteobacteria ↓	Burkholderiales ↓	Alcaligenaceae ↓	Parasutterella ↓
	Deltaproteobacteria ↓	Desulfovibrionales ↓	Desulfovibrionaceae ↓	Desulfovibrio ↓
	Gammaproteobacteria	Enterobacteriales ↑	Enterobacteriaceae ↑	Enterobacter ↑
				Serratia ↑
		Pasteuralles ↓	Pasturellaceae ↓	
Tenericutes ↓	Mollicutes ↓	Mollicutes.RF9 ↓	Mollicutes.RF9 ↓	O_Mollicutes ↓
		NB1.n ↓		O_NB1.n ↓
Verrucomicrobia ↓	Verrucomicrobiae ↓	Verrucomicrobiales ↓	Verrucomicrobiaceae ↓	Akkermansia ↓

In Table S1, arrows indicate if relative abundance has increased (↑) or decreased (↓). Taxa without arrows describe phylogeny and demonstrated no significant difference in abundance. Taxa with significant differences (q < 0.05) in distribution between CF and HC groups were calculated using ANCOM analysis.

Table S2. Bacterial taxa at each taxonomic rank, which demonstrate significantly different abundance in the cystic fibrosis gut longitudinally.

Phylum	Class	Order	Family	Genus
Bacteroidetes ↓	Bacteroidia ↓	Bacteroidales ↓	Bacteroidaceae	Bacteroides ↓
Deinococcus. Thermus				
Firmicutes				Tyzzerella 4 ↓
Verrucomicrobia	Verrucomicrobiae			

In Table S2, arrows indicate if relative abundance has increased (↑) or decreased (↓). Taxa without arrows describe phylogeny and demonstrated no significant difference in abundance. Taxa with significant differences (q < 0.05) in distribution between CF and HC groups were calculated using ANCOM analysis.

Table S3. Correlations between selected dietary metrics and clbB prevalence in cross-sectional CF analysis.

	Mean (SD)	*p*-Value
**Fat**		
clbB positive	11.2 (1.3)	0.6
clbB negative	10.8 (1.7)	
**Saturated Fats**		
clbB positive	4.6 (0.6)	1.0
clbB negative	4.6 (0.98)	
**Processed Meat**		
clbB positive	92 (48)	0.9
clbB negative	91 (50)	
**Total Sugar**		
clbB positive	11 (3.1))	0.6
clbB negative	12 (3.2	
**Total Fibre**		
clbB positive	2.6 (0.55)	0.5
clbB negative	2.5 (0.77)	

In Table S3, mean and p-value following Student's *t*-test and Mann–Whitney U test analysis of correlations between clbB prevalence and selected dietary metrics, total fat, saturated fat, processed meat, total sugar, and total fibre. The distribution of data was assessed with the use of the Shapiro–Wilk test for normalcy.

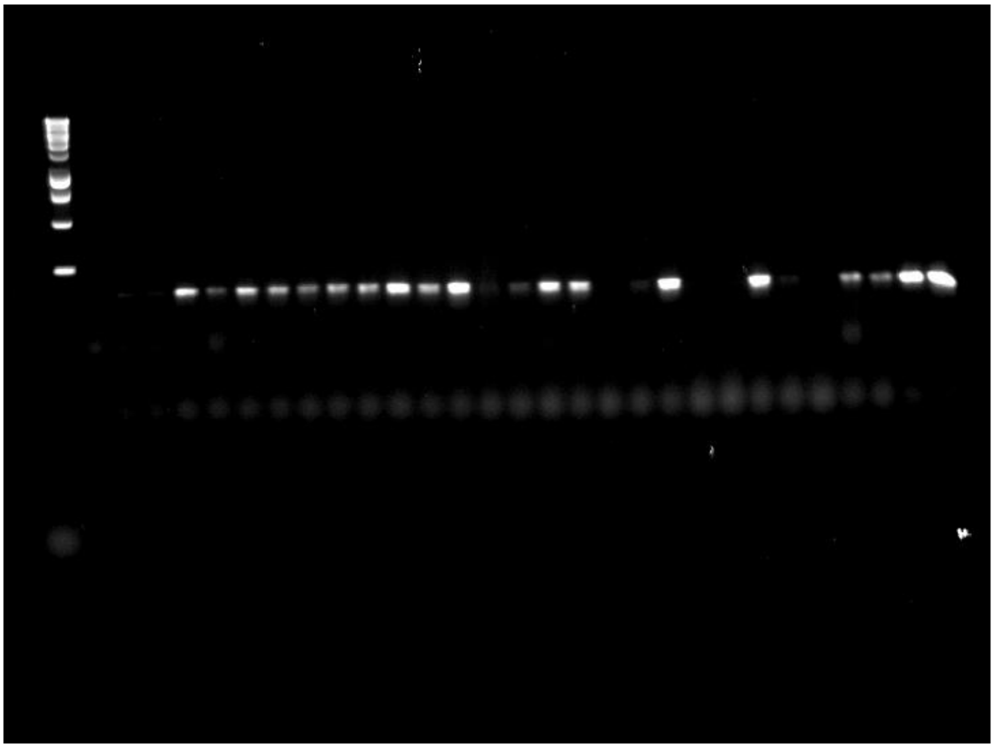

Figure S1. PCR products following agarose gel electrophoresis for detection of clbB in a sample of CF patients.

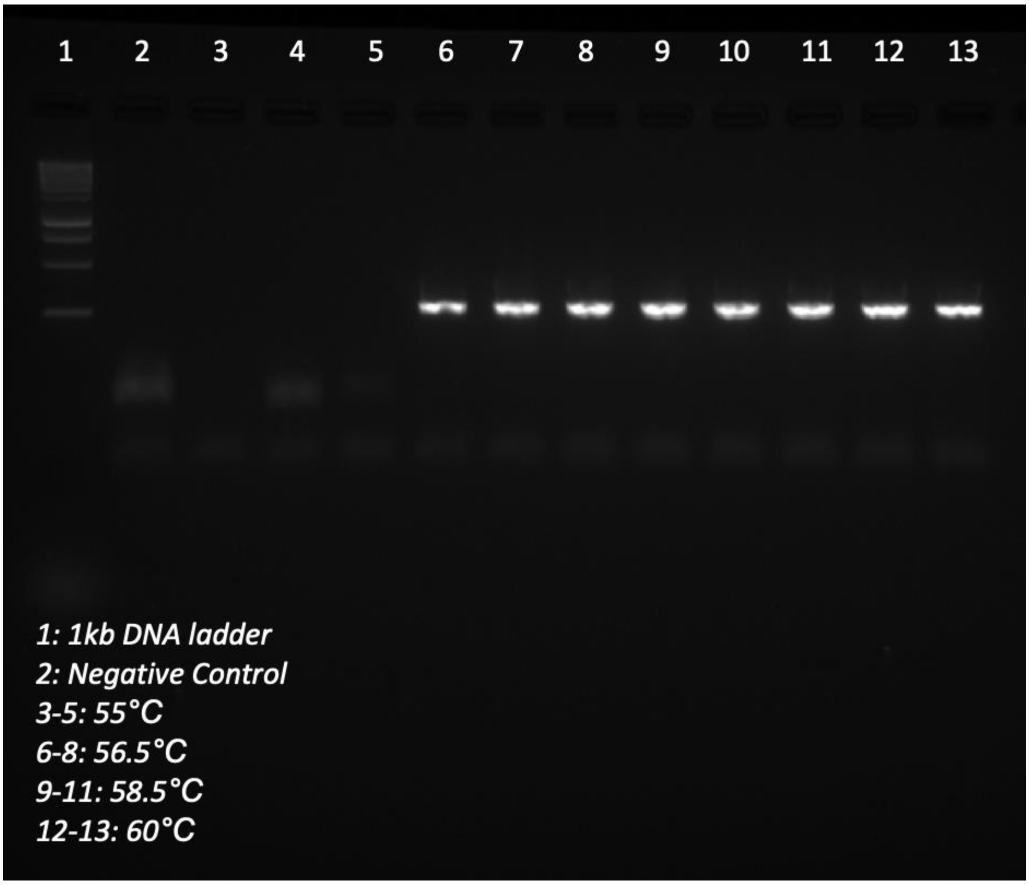

Figure S2. PCR products following agarose gel electrophoresis for optimisation of clbB gene primers. In total 11 PCR reactions were prepared with our pks+ *E. coli* positive control, isolated from Mutaflor® Escherichia coli strain Nissle 1917 (Mutaflor), along with one negative control prepared with nuclease free water. PCR reactions were amplified with forward and reverse clbB primers in Invitrogen® Super Mix. A 1 kB DNA ladder was run alongside samples to ensure the correct product was obtained. Reaction tubes were amplified across 4 different annealing temperatures (3 × 55 °C, 3 × 56.5 °C, 3 × 58.5 °C, and 2 × 60 °C) using four SimpliAmp™ Thermal Cyclers (Applied Biosystems/Thermo Fisher Scientific, Waltham, MA, USA) set at each temperature. From the result, a determination on the optimal annealing temperature (58.5 °C) was made. PCR = polymerase chain reaction, pks+ *E. coli* = polyketide synthase-positive *Escherichia coli*, DNA = deoxy-ribonucleic acid.

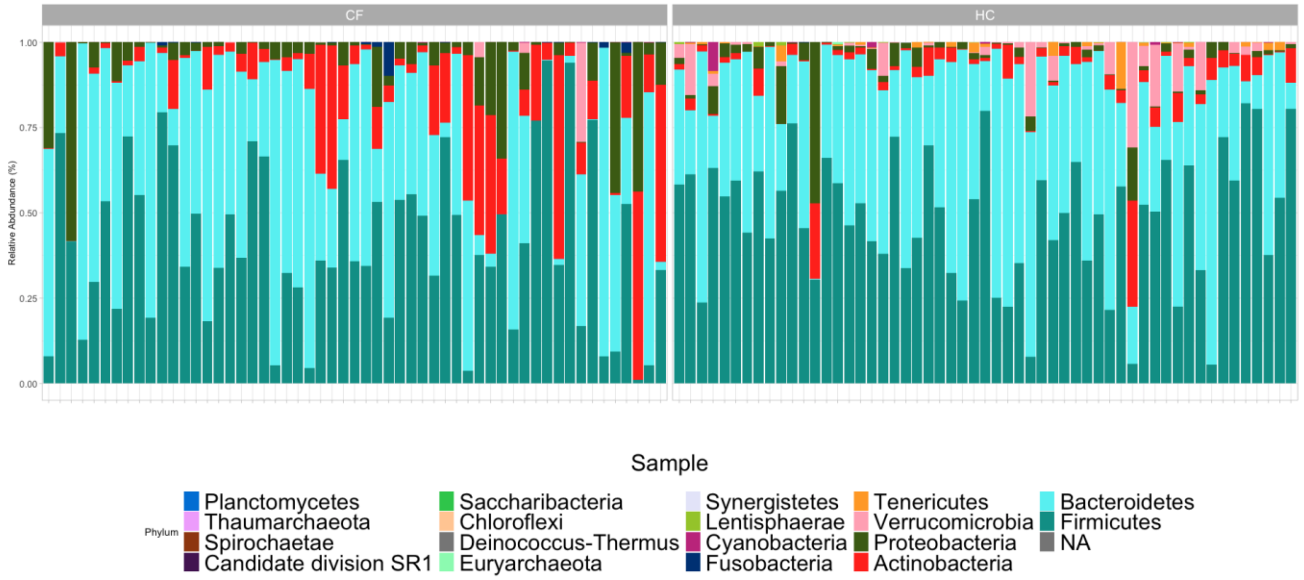

Figure S3. Relative abundance (%) of the most abundant bacterial phyla in the cystic fibrosis gut versus healthy controls.

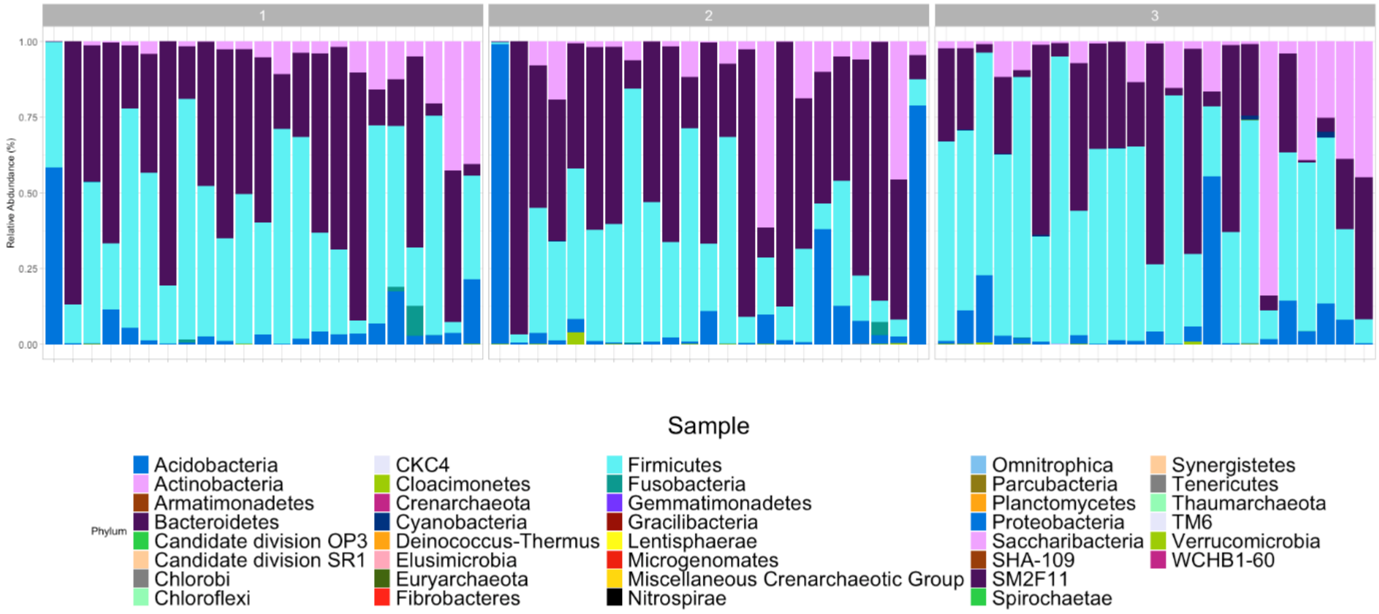

Figure S4. Relative abundance (%) of the most abundant bacterial phyla in the cystic fibrosis gut over time.

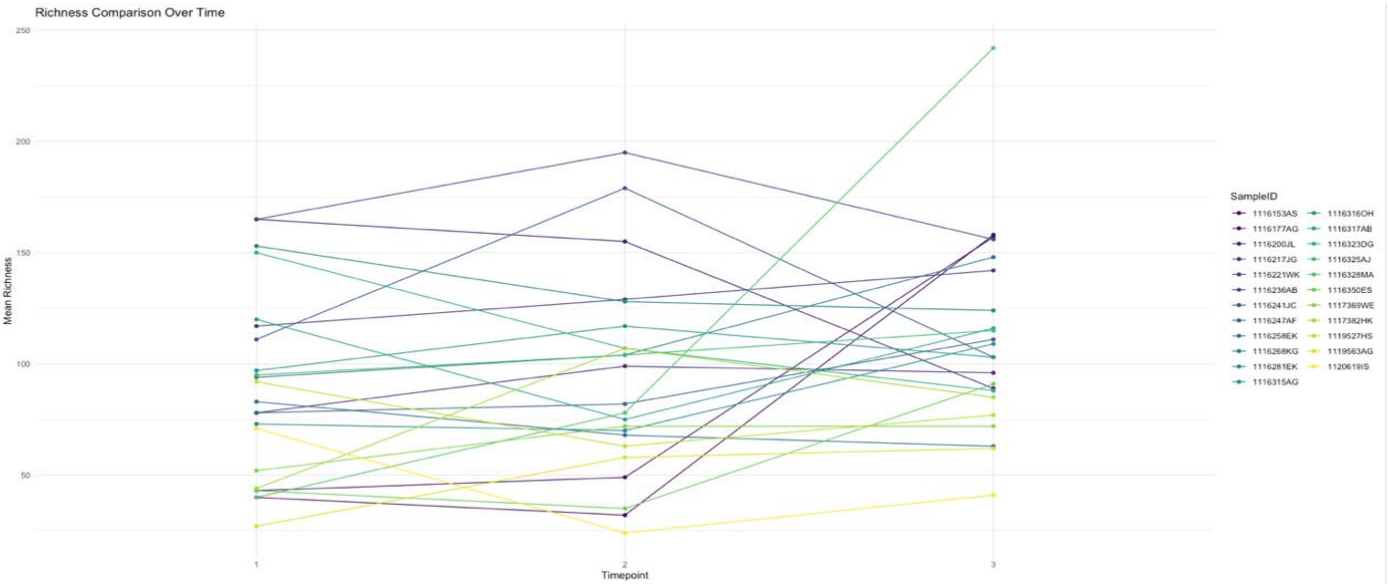

Figure S5. Longitudinal microbial richness of cystic fibrosis patients.

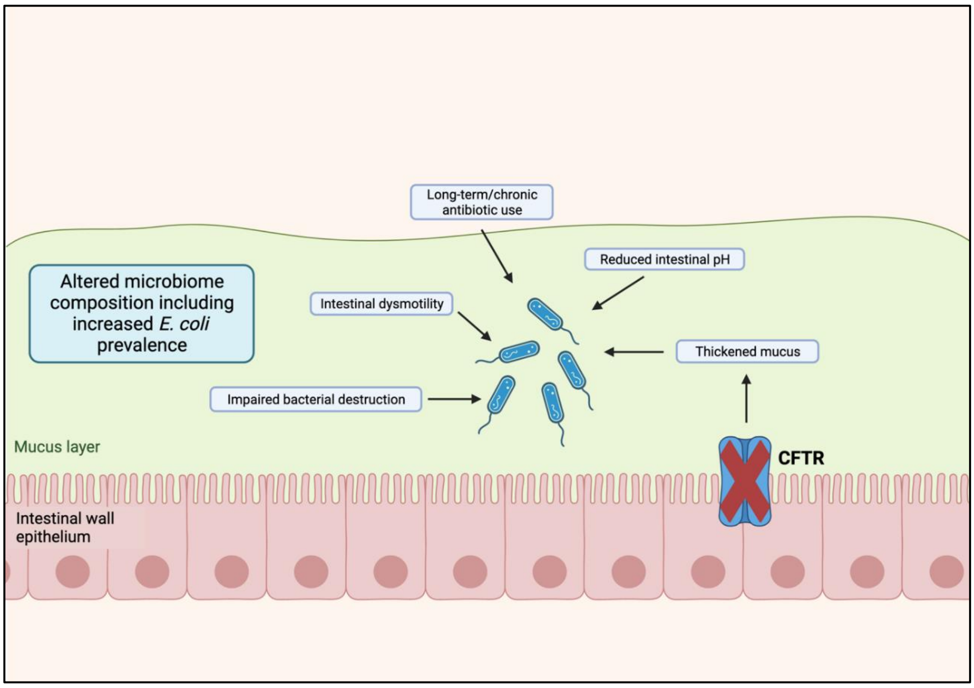

Figure S6. The proposed pathogenic factors associated with the development of an altered gastrointestinal microbiota in cystic fibrosis. The composition of the gut microbiota in cystic fibrosis is abnormal, where CFTR dysfunction contributes to reduced bicarbonate secretion and subsequent reductions in pH. Furthermore, reduced bicarbonate contributes to the development of hyper-viscous mucus, impairing intestinal motility. Regular antibiotics use and a diminished innate immune response against pathogenic bacteria may also contribute. The cumulative impact of these changes is an altered gastrointestinal milieu is characterised most notably the raised abundance of Escherichia coli. CFTR = cystic fibrosis transmembrane conductance regulator. *E. coli* = Escherichia coli. Adapted from "Epithelial Mucosa", by BioRender.com (2023). Retrieved from https://app.biorender.com/biorender-templates.

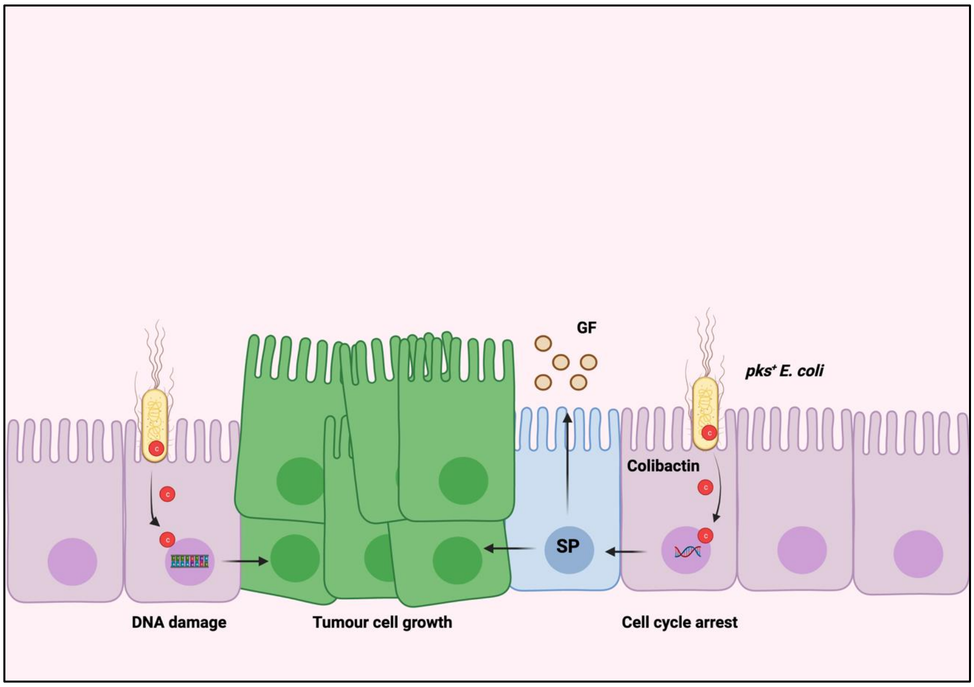

Figure S7. Colibactin induces colon cell mutation. During infection with pks⁺ *E. coli*, where pks⁺ *E. coli* comes into direct contact with the host epithelium, colibactin molecules migrate to the host cell nucleus to initiate DNA breaks. Infection with pks⁺ E. coli also facilitates cell-cycle arrest, causing a senescence related secretory phenotype, which stimulates growth factor release into the tumour microenvironment [20]. These combined processes contribute to colon cell mutagenesis. pks⁺ *E. coli* = polyketide synthase-positive Escherichia coli, DNA = deoxy-ribonucleic acid, SP = senescence-associated secretory phenotype, GF = growth factor. "Created with BioRender.com.".

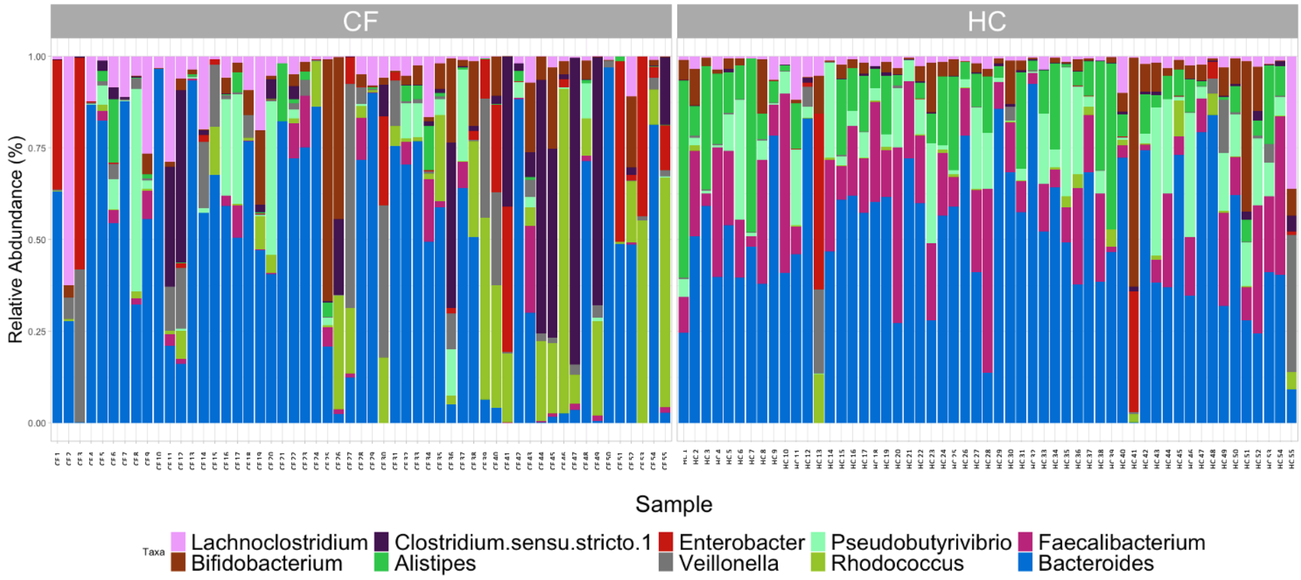

Figure S8. Relative abundance (%) of the top 10 most abundant bacterial genera in the cystic fibrosis gut versus healthy controls. Samples have been de-identified and assigned an ID based on diagnosis (CF1–CF55, HC 1–55). The diagnosis of each sample is indicated above its respective column. Relative abundance was calculated as the abundance of a single zOTU, divided by the sum of total zOTU abundances. The Nucleotide Basic Local Alignment Search Tool (BLASTn) in the SILVA 123 ribosomal RNA (rRNA) database was utilised to align zOTUs to specific genera. This graph was generated using ggplot2 in R [33]. Relative abundance = the rarity of a particular species relative to other species within the community, CF = cystic fibrosis, HC = healthy control, zOTU = zero-distance operation taxonomic unit.

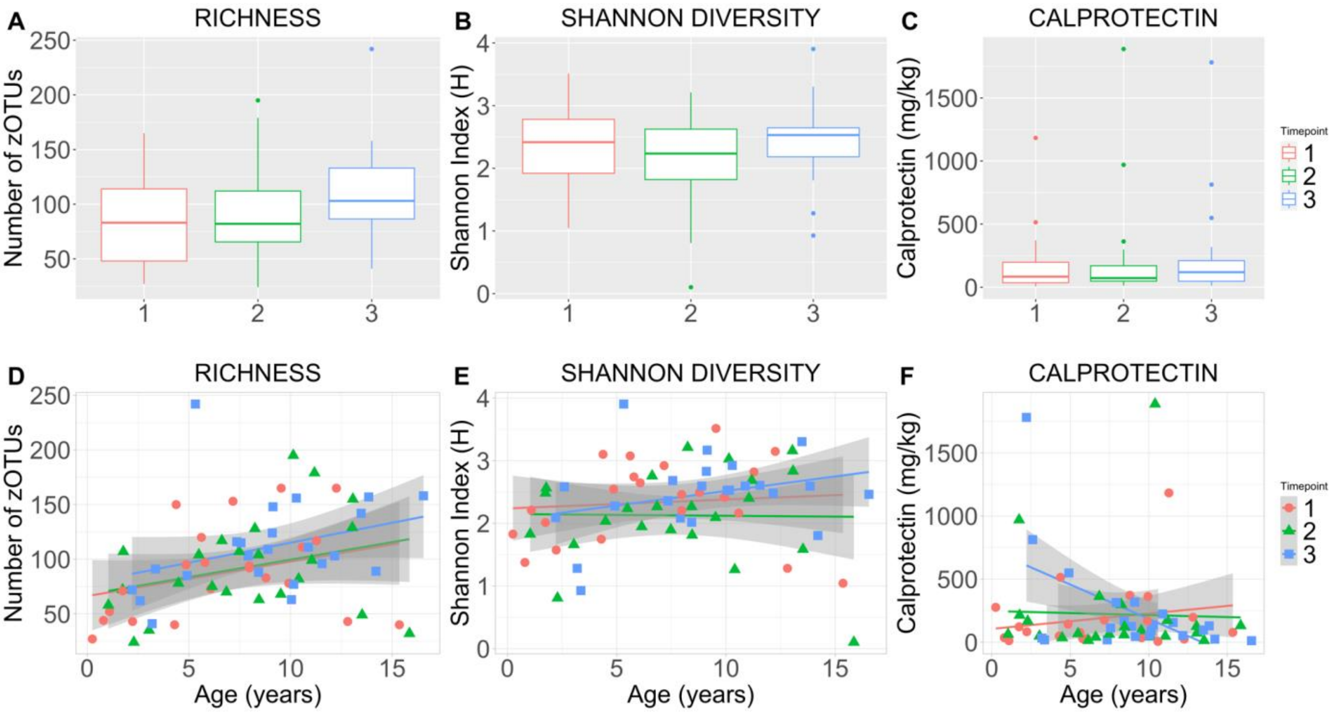

Figure S9. Longitudinal analysis of alpha diversity indices and fecal calprotectin in children with cystic fibrosis. Boxplots depict fecal sample richness (number of zOTUs) (**A**), Shannon diversity (Shannon Index (H)) (**B**), and the concentration of fecal calprotectin (mg/kg) (**C**) across 3 consecutive timepoints, spaced roughly 6 months apart. Scatterplots illustrate sample richness (**D**), Shannon diversity (**E**), and fecal calprotectin (**F**) across timepoints with respect to age (years). Coloured lines indicate the cohort means, while shaded regions indicate 95% confidence intervals constructed from generalised linear models (**D**–**F**). calprotectin = a measure of intestinal inflammation, zOTU = zero-distance operation taxonomic unit.

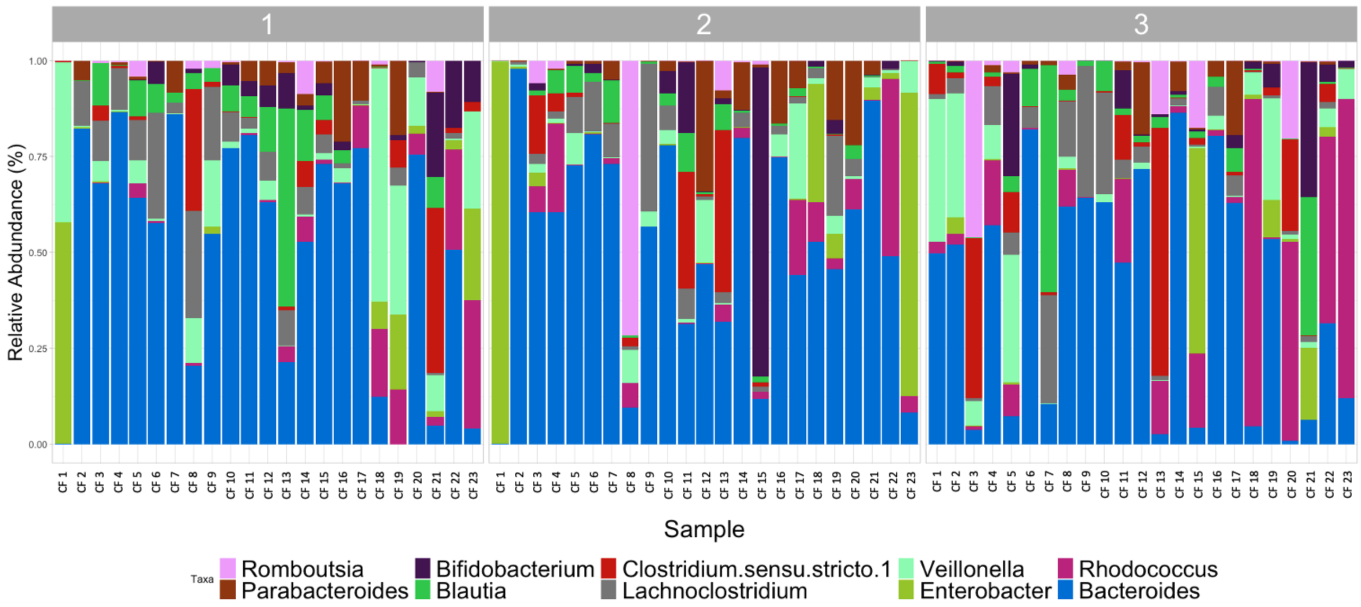

Figure S10. Longitudinal analysis of relative abundance for the 10 most abundant bacterial genera in cystic fibrosis patients. Samples have been de-identified and assigned an ID (CF1–CF23). Samples with the same ID correspond to the same patient. The timepoint of each sample is indicated above its respective column. Relative abundance was calculated as the abundance of a single zOTU divided by the sum of total zOTU abundances. The Nucleotide Basic Local Alignment Search Tool (BLASTn) in the SILVA 123 ribosomal RNA (rRNA) database was utilised to align zOTUs to specific genera. This graph was generated using ggplot2 in R [33]. Relative abundance = the rarity of a particular species relative to other species within the community, CF = cystic fibrosis, zOTU = zero-distance operation taxonomic unit.

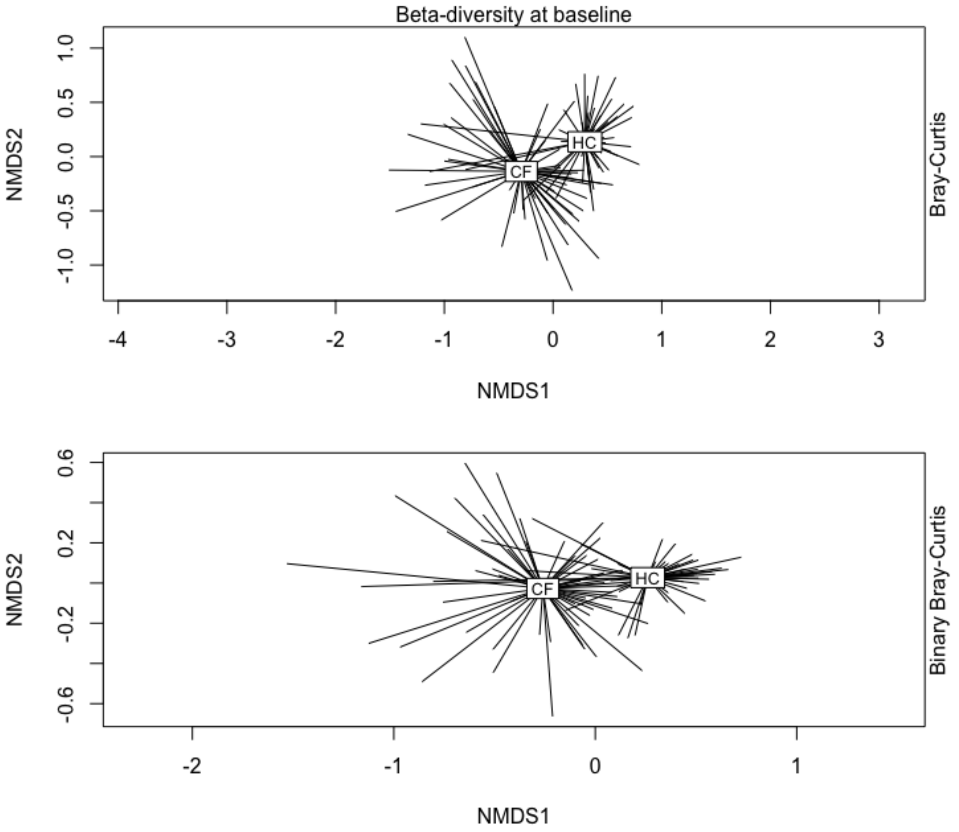

Figure S11. Bray–Curtis and binary Bray–Curtis analysis for cross-sectional comparison of children with cystic fibrosis versus healthy controls.

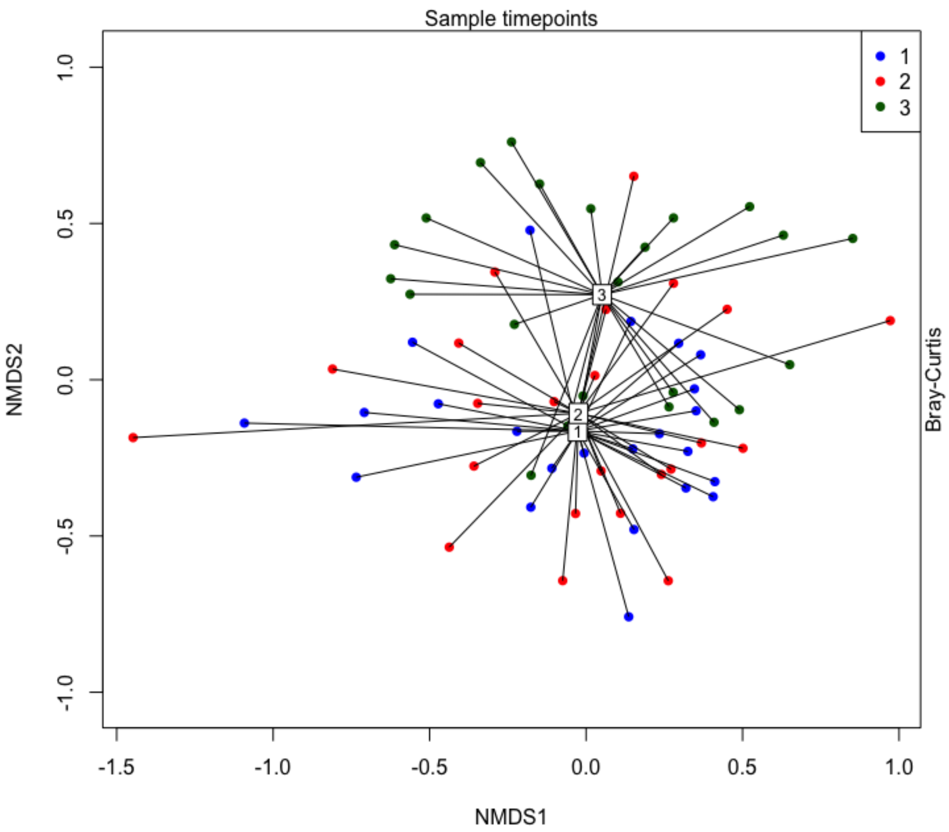

Figure S12. Bray–Curtis analysis for longitudinal comparison of children with cystic fibrosis at first, second, and third sample timepoints.
